# Derivation of New Threshold of Toxicological Concern Values for Exposure via Inhalation for Environmentally-Relevant Chemicals

**DOI:** 10.3389/ftox.2020.580347

**Published:** 2020-10-16

**Authors:** Mark D. Nelms, Grace Patlewicz

**Affiliations:** ^1^Oak Ridge Institute for Science and Education, Oak Ridge, TN, United States; ^2^Center for Computational Toxicology & Exposure (CCTE), U.S. Environmental Protection Agency, Research Triangle Park, Durham, NC, United States

**Keywords:** threshold of toxicological concern (TTC), inhalation, Cramer, Kroes, Verhaar, OASIS Aquatic MOA

## Abstract

The requirements of amended Toxic Substances Control Act (TSCA) stipulates that the US Environmental Protection Agency (US EPA) evaluate existing chemicals and make risk based assessments. There are ~33,000 substances that are active in commerce on the TSCA public non-confidential inventory, many of which lack available toxicity and exposure information to inform risk-based decision making. One approach to facilitate the assessment of these substances being considered is the Threshold of Toxicological Concern (TTC). TTC values are intended to identify safe levels of exposure for data poor substances. TTC values derived based on non-cancer data notably by Munro et al. (1996) are well-established and are in routine use for food additive applications however far less attention has been focused on developing TTC values where inhalation is the route of exposure. Here, an effort was made to derive new inhalation TTC values using the EPA's Toxicity Values database, ToxValDB. A total of 4,703 substances captured in ToxValDB were assigned into their respective TTC categories using the Kroes module within the Toxtree software tool and custom profilers developed in Nelms et al. (2019) and Patlewicz et al. (2018). For the substances assigned into the 3 Cramer classes, the 5th percentiles were calculated from the empirical cumulative distributions of No observed (adverse) effect level (concentration) values. The 5th percentiles were converted to their respective TTC values and compared with published values reported by Escher et al. (2010) and Carthew et al. (2009). The TTC values derived from ToxValDB were orders of magnitude more conservative, further, Cramer classification was not found to be effective at discriminating potencies. Instead, use of aquatic toxicity modes of action such as Verhaar et al. (1992) were found to be effective at separating substances in terms of their potencies and new TTC thresholds were derived.

## Introduction

The basis of the Threshold of Toxicological Concern (TTC) approach relies on setting a level of human intake or exposure that is considered to be of negligible risk, despite the absence of chemical-specific toxicity data. TTC is a pragmatic approach to assess the safety of substances where exposure is estimated to be very low (Barlow, 2005). Established TTC values that are used in practice have been derived from grouping experimental toxicity data from animal studies based on structural chemistry considerations. A comprehensive review of the history of the TTC concept and its application has been described by Barlow (2005) as part of the ILSI monograph as well as guidance by EFSA/WHO (2016), Hartung (2017), and EFSA Scientific Committee (2019). The approach is well-established in evaluating substances on a case by case basis for food additives, food contact, and flavoring ingredient applications (Kroes et al., 2004; EFSA/WHO, 2016).

The practical application of TTC follows a tiered decision tree (Kroes et al., 2004) where different thresholds spanning several orders of magnitude are used for different tiers of chemicals. Some chemicals are excluded from the TTC because they are not represented in the underlying toxicity datasets supporting TTC or because standard risk assessment approaches are more appropriate. The workflow in practice starts with a consideration of whether a substance should be excluded from the TTC approach, that it is to say, it is inorganic, bioaccumulative (dioxin like), a protein, a polymer etc. If substance is not excluded from consideration for TTC, the next step in the workflow considers whether the substance presents any structural alerts that raise concern for potential genotoxicity. There are a set of 5 high potency carcinogens—known as the “cohort of concern” that render substances not suitable for the TTC approach; namely azoxy compounds, nitroso compounds, aflatoxin-like, steroids, and 2,3,7,8-dibenzo-p-dioxin and its analogs. If a substance presents a genotoxicity alert but it is not an alert captured by the “cohort of concern” than a TTC value of 0.15 ug/day (0.0025 ug/kg-day), the most conservative value is assigned. If a substance presents no alerts for genotoxicity, the next step in the TTC workflow considers whether the substance is an organophosphate or carbamate—if yes, then a TTC value of 18 ug/day (0.3 ug/kg-day) is assigned. If the substance is not an organophosphate/carbamate than it is assigned into one of the 3 Cramer structural classes (Cramer et al., 1978), where Class III has a TTC value of 90 ug/day (1.5 ug/kg-day), Class II a value of 540 ug/day (9 ug/kg-day), and Class I, 1800 ug/day (30 ug/kg-day). The TTC thresholds for the 3 Cramer classes were calculated from the distribution of No observed (adverse) effect levels (NO(A)ELs) using a database of 613 chemicals with 2941 NO(A)ELs developed by Munro et al. (1996). For each of the 613 chemicals, the most conservative NOAEL was selected, based on the most sensitive species, sex, and endpoint. The 5th percentile NOAEL was calculated for each structural classes and converted to an intake followed by the application of a 100-fold uncertainty factor. The Cramer class assignment is based on a set of 33 questions covering reactivity and recognized pathways for metabolic deactivation to rank expected levels of oral systemic toxicity (Cramer et al., 1978). The Cramer class decision tree and the Kroes et al. (2004) workflow has been implemented in software tools such as Toxtree (Patlewicz et al., 2008) and the OECD Toolbox (Schultz et al., 2018).

There have been many refinements and evaluation for TTC values either to augment the underlying database or verify its relevancy for food (Reilly et al., 2019) as well-other sectors such as cosmetics (Yang et al., 2017), environmental chemicals (Nelms et al., 2019), fragrances (Patel et al., 2020), and antimicrobials (Yang et al., 2020). Whilst application of TTC is usually conducted on an individual chemical basis, Patlewicz et al. (2018) outlined an alternative approach of applying TTC as part of a risk-based prioritization to evaluate thousands of substances. The approach relied upon predicted exposures using a general high-throughput toxicokinetic model (Wambaugh et al., 2014) coupled with non-cancer TTC values for oral exposures (Kroes et al., 2004) to assign substances into bins of lower or higher concern.

For all the efforts on evaluating and applying TTCs for oral exposure, far less attention has been paid to deriving TTC values for the inhalation route of exposure. Past efforts in particular include seminal work by Carthew et al. (2009), and Escher et al. (2010). More recent efforts include assessments to derive TTCs from Derived No Effect Levels (DNELs) from Hoersch et al. (2018) and Occupational Exposure Limits (OELs) from Chebekoue and Krishnan (2017).

Carthew et al. (2009) compiled inhalation toxicity studies for 92 substances to derive TTC values for local and systemic effects that would be impactful to evaluate ingredients used in consumer aerosol products. The studies evaluated used were sourced from the EPA High Production Volume Chemical reports and through published evaluations from EU Member State Authorities such as BfR (German Federal Institute for Risk Assessment) as well Chemical Industry through ECETOC (European Center for Ecotoxicity and Toxicology of Chemicals). Studies were reviewed to exclude substances that would not be applicable for TTC or not relevant for aerosol consumer products. Substances that were genotoxic carcinogens or *in vivo* mutagens were also excluded. A total of 92 studies were reviewed. Substances were then profiled using the Toxtree tool (Patlewicz et al., 2008) to assign Cramer structural classes. Only 4 substances were assigned to Cramer structural class II, hence the remaining TTC derivations were conducted on Cramer I and III class substances only. Local and systemic TTCs were derived for individual Cramer classes and for all substances by application of a 25-fold uncertainty factor to the 5th percentiles of the distributions of No observed (adverse) effect concentrations (NOAECs) and NOAELs. The proposed systemic TTC values were 980 ug/person per day for substances in Cramer class I and 170 ug/person per day for substances in Cramer class III, whereas the proposed local TTC values were 1,400 ug/person and 470 ug/person for Cramer class I and III, respectively.

The RepDose database of the Fraunhofer ITEM, Germany was used in Escher et al. (2010) to derive TTC values for substances in the Cramer classes in a manner close to what Munro et al. (1996) had described for oral TTCs. The 5th percentiles of NOEC values in ppm or mg/m^3^ were used to determine inhalation TTC values. Escher et al. (2010) also attempted to evaluate how local and systemic toxicity influenced the TTC values derived. Local and systemic NOEC values were identified and target organs at study LOAEC were analyzed. TTC values were also derived after removal of substances that were organophosphates or presented with structural alerts for genotoxicity. The TTC values for systemic effects were reported as 71 ug/day for Cramer class I, 10 ug/day for class II and 4 ug/day for class III substances.

In this study, an effort was made to investigate the feasibility of proposing new TTC values for inhalation using data from ToxValDB that had been previously utilized in Nelms et al. (2019) and comparing them against the published values reported by Carthew et al. (2009) and Escher et al. (2010). These two studies were selected since they were ones where the underlying data in terms of points of departure were comparable and where the underlying data was provided to allow a reproduction of the TTC derived to even be attempted.

The steps performed in this study can be summarized as follows:

1. Gather the chemicals and summary toxicity study data from ToxValDB.
2. Identify the chemical structures for all the chemicals in ToxValDB.
3. Process the chemicals through the Kroes et al. (2004) workflow, but using the adaptations in Patlewicz et al. (2018) and the modified organophosphate and carbamate alerts established in Nelms et al. (2019) since these facilitated batch processing.
4. For substances that were assigned as belonging to the 3 Cramer classes, filter ToxValDB to identify relevant studies that met the same criteria as used by Munro et al. (1996) but where the route of exposure was inhalation.
5. Remove statistical outliers and taking the minimum NOAEL/NOAEC for each chemical as the “representative” value (in either mg/m^3^ or ppm units), and deriving the 5th percentile values.
6. Compare the 5th percentile and their associated TTC values to those published by Carthew et al. (2009) and Escher et al. (2010).
7. Explore other means to categorize the substances beyond Cramer designations if required and propose new TTC values.

Regarding (7), approaches to categorize focused on using aquatic mode of action assignments such as those by Verhaar et al. (1992). This was motivated by work by Veith et al. (2009) who found that whilst fish and mammalian inhalation baseline toxicity was not directly comparable because the external media are different, the blood thermodynamic activity for LC50 (narcosis) was the same. At steady state, the activity in air/water equals the activity in blood by definition:


$$\begin{array}{l} Activity=C\times y \end{array}$$

where C is concentration and y is an activity coefficient.

The thermodynamic activity at any concentration could be estimated by dividing by the solubility in the medium such that the activity for narcosis in fish was the LC50 fish divided by water solubility and the activity for narcosis in rat was the LC50 inhalation in rat divided by air solubility. If the activity for narcosis in fish and rat were equal the plot of LC50 vs. solubility in exposure medium should be the same [as shown on slide 10 in Veith et al. (2006)].

Veith et al. (2009) found this to be the case and derived a baseline model to predict LC50 in acute inhalation studies in rodents using the logVapour Pressure for neutral organic substances—i.e., those substances that contain no overt functional groups indicative of electrophilic reactivity. For substances with electrophilic features, using *in chemico* reactivity data was found to be a good predictor of acute inhalation toxicity. Thus, categorizing substances into modes of action (MOA) that have been characterized for aquatic effects might prove effective for discriminating and subcategorizing substances for their systemic inhalation toxicity.

## Materials and Methods

### Toxicity Data Sources

Three sources of toxicity data were utilized in this study: (1) the US EPA's Toxicity Values database (version 7), referred to as ToxValDB; (2) the inhalation TTC dataset from Appendix A of the Escher et al. (2010) manuscript, referred to as the “Escher dataset”; and (3) the inhalation TTC dataset from Appendix A of the Carthew et al. (2009) manuscript, referred to as the “Carthew dataset.”

#### Toxicity Value Database (ToxValDB)

ToxValDB consists of a collection of summary level *in vivo* test data from a variety of study types typically used in risk assessments. It comprises point of departure (POD) values such as NO(A)EC and lowest-observed (adverse) effect concentration (LO(A)EC) data. These data have been aggregated from over 40 publicly available sources including US Federal and State agencies [e.g., US EPA, US Food and Drug Administration (FDA), and California EPA], alongside international organizations [e.g., World Health Organization (WHO)], as well as data submitted under regulatory frameworks, such as the European Union's REACH regulation [e.g., non-confidential registration data submitted to the European Chemicals Agency (ECHA) by industry registrants].

#### Carthew and Escher Datasets

The Carthew dataset consists of local no-observed adverse effect concentration (NOAEC) values (in mg/m^3^) and systemic no-observed adverse effect level (NOAEL) values (in mg/kg-day) for 92 chemicals. These data were extracted from publicly available reports and published evaluations from a number of agencies and organizations, such as US EPA and the German Federal Institute for Risk Assessment (BfR).

The Escher dataset comprises a combination of local and/or systemic and general no-observed effect concentration (NOEC) values (all in ppm) for 203 chemicals from RepDose [www.fraunhofer-repdose.de, Bitsch et al. (2006)]. The general NOEC for each chemical within the Escher dataset is the lower of either the local or systemic NOEC value: where only a systemic NOEC is present (in 198 cases), this is also the general NOEC. Cramer class assignments were reported in both datasets. Both the Carthew and Escher datasets were retrieved from the respective journal articles and, after making minor adjustments, converted into individual tab separated value (tsv) files for subsequent data analysis.

The processed datafiles for both the Carthew and Escher datasets are provided as Supplementary Information.

### Chemical Structure Data: ToxValDB

Defined chemical structures (such as SMILES: Simplified Molecular-Input Line-Entry System) were required in order to profile the chemicals present in ToxValDB through the TTC decision tree within Toxtree version 3.1. Using the DSSTox substance identifiers (DTXSID), QSAR-ready SMILES strings, ToxPrint chemical fingerprints (Yang et al., 2015), and average mass information [Molecular Weight (MW)] were extracted from the US EPA's CompTox Chemicals Dashboard, herein referred to as the Dashboard [https://comptox.epa.gov/dashboard, Williams et al. (2017), Grulke et al. (2019)]. QSAR-ready SMILES make reference to a standardization procedure for chemical structures as described in Mansouri et al. (2018) and Mansouri (2017). The procedure serves to remove salt counterions, remove stereochemistry, standardize tautomers and nitro groups, correct valences and neutralize structures when possible. Of the 15,960 unique substances present in ToxValDB, QSAR-ready SMILES were available for 4,703 chemicals (see notebook_01).

### Chemical Structure Data: Carthew and Escher Datasets

The same information was queried for the substances in the Carthew and Escher datasets. To retrieve this information, either the chemical name (Carthew dataset) or Chemical Abstract Service (CAS) number (Escher dataset) were utilized as search terms.

After performing a batch search of the Dashboard with the Carthew dataset, only two chemicals (hydroxypropyl acetate and mineral oils) were present without SMILES information. There were 27 instances where a substance could not be identified based upon the name provided. For approximately half of the 27 substances, the issues in identification typically arose due to a second name or abbreviation being present in parentheses at the end of the substance name. In these instances, both names were used to re-search the Dashboard and compared against the synonyms present in the Dashboard. Where both names matched the same substance, only one of the names was retained: preferentially retaining a synonym designated as “valid” in the Dashboard. If a substance still could not be found within the Dashboard, ChemSpider (http://www.chemspider.com) and ECHA (http://echa.europa.eu) were used to search for synonyms, which were then used as the search term within the Dashboard. When a search on the substance name or a synonym returned an ill-defined substance in the Dashboard, ChemSpider was used to retrieve the average mass. After performing these searches, average mass information was retrieved for all but 3 substances and QSAR-ready SMILES for all but 13 substances. The substances for which information could not be retrieved were typically for mixtures, polymers, or inorganics (specific examples include mineral oils, benzene alkylate 225, PEG-200, hydrochloric acid, hydrogen peroxide, silica, sulfuric acid).

After searching the identifiers in the Escher dataset using the Dashboard, data was retrieved for all but two substances: diphenylmethane diisocyanate and dipropylene glycol monomethyl ether. The CAS numbers of these were searched within ChemSpider and the resulting names were compared with that in the Escher dataset; where there was a match, the average mass was extracted. By doing this, average mass information could be retrieved for every chemical in the Escher dataset and QSAR-ready SMILES for all but the 2 previously named chemicals.

### Profiling of Substances Through the Kroes et al. (2004) Workflow

The 4,703 substances within ToxValDB, for which QSAR-ready SMILES were available, were profiled through Toxtree (v3.1.0) (IdeaConsult, Ltd) in order to assign them into the appropriate TTC category. This was carried out using two of the original modules, namely the Cramer rules (original) (Patlewicz et al., 2008) and the Kroes TTC module. Additionally, these substances were also profiled using 3 custom modules developed *ad hoc* by Nelms et al. (2019) and Patlewicz et al. (2018) intended to identify carbamates, organophosphates (OPs), and steroids, respectively. These custom profilers in Patlewicz et al. (2018) were utilized since the Kroes workflow within Toxtree is not designed for batch processing without providing exposure information upfront. The custom modules allowed for the Kroes workflow to be replicated. The custom profilers developed in Nelms et al. (2019) ensured organophosphates to be more specifically identified rather than rely on broad structural feature definitions for organophosphates as implemented in the Toxtree Kroes workflow implementation.

The Cramer structural class assignments as provided in Carthew et al. (2009) and Escher et al. (2010) were used rather than attempting to profile the substances *de novo*.

### Annotation and Extraction of Relevant Data From ToxValDB

#### Annotation of Additional Study Information

Study data were initially identified within ToxValDB that were either subacute, subchronic, chronic, reproductive, developmental, or multigenerational study type. This was carried out by creating a new field (i.e., study_length) to designate chronic, subchronic, and reproductive studies on the basis of reported study duration and study type information. A short-term/repeat dose study was considered to be chronic if the “study_type” column stated it was a chronic study or if the study duration was over 100 days (or week/month equivalent). Similarly, a study was considered to be subchronic if the “study_type” column stated it was a subchronic study or if the study duration was >=35 days and < 100 days (or week/month equivalent). On the other hand, a short-term/repeat dose study was only considered to be a reproductive study if the “study_type” column stated as such.

#### Extraction of Relevant Inhalation Study Information

As the dose measurements for each study were provided in either ppm- or mg/m^3^-related units, toxicity values were converted into common units. Toxicity values in g/m^3^ and ug/m^3^ were converted into mg/m^3^, no transformation was required between mg/L and mg/m^3^ as they are equivalent. Equation (1) was utilized to convert the toxicity values between ppm and mg/m^3^ and, conversely, from mg/m^3^ to ppm.


(1) $$\begin{array}{l} NOEC\;\left(mg/m^{3}\right)=\frac{NOEC\left(ppm\right)\times MW\left(\frac{g}{mol}\right)}{24.45\left(\frac{l}{mol}\right)} \end{array}$$

where MW is the molecular weight of the chemical and NOEC is the NO(A)EL or NO(A)EC of the chemical in either ppm or mg/m^3^.

New columns with standardized units were created to capture ppm and mg/m^3^ units separately. ToxValDB records were then filtered to remove ambiguous records, i.e., those with a toxicity value of 0 or those with a value of −999. New columns were created to capture toxicity values adjusted on the basis of duration of exposure—this allowed for subchronic and subacute values to be used in the analysis.

Rodent and rabbit species names were standardized so that these could be readily selected for filtering. In this case, records where rat, mice, rabbits, and partial names were identified in the set of studies and mapped to a generic common species tag of rodent. The dataset was filtered to select (a) study length as subacute, subchronic, repeat dose, chronic, reproductive, developmental, and multigenerational; (b) exposure route as inhalation, (c) toxval type as NO(A)EL or NO(A)EC point of departure; and (d) species as “rodent” (which covered rats, mice, and rabbits).

### Derivation of the TTC Values for the Cramer Structural Classes Using ToxValDB

**Figure 1:**
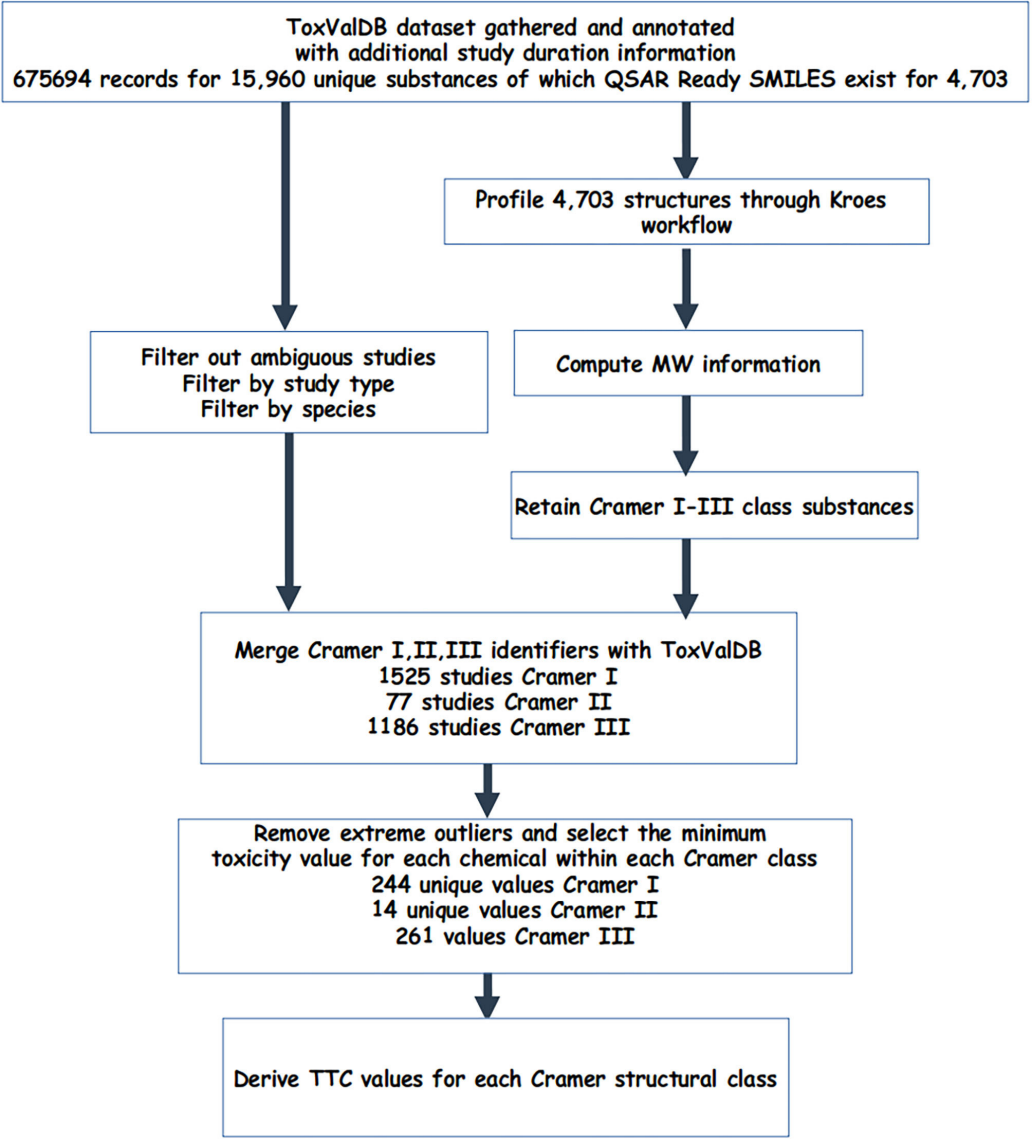
Workflow to describe the creation of the datasets used.

ToxValDB data was then merged with the 3 Cramer structural classes and were processed further as follows: (1) for substances with only 1 study, this was retained; (2) for substances with more than 1 study, extreme outliers, i.e., statistical outliers that exceeded ×1.5 the interquartile range were removed (Tukey, 1977) and the minimum value was returned. This was carried out for both units, mg/m^3^ and ppm. Figure 1 describes the workflow to generate the Cramer class datasets.

In the original TTC derivations for oral exposures by Munro et al. (1996), NOAEL values were found to fit a log normal distribution and the 5th percentile was computed from the fitted distribution rather than from the empirical distribution. Here, the empirical cumulative distributions (ECDFs) were first plotted for all three Cramer structural classes after transforming their NOAEC/NOAEL values into log10 equivalents.

To explore whether the ECDFs approximated normal distributions, the cumulative distribution function (CDF) for a normal distribution for each Cramer class was overlaid on the empirical CDFs plot. If the generative distribution for each Cramer structural class were normally distributed, then the ECDF and CDF plots should closely match each other. CDFs were plotted by computing the mean and standard deviation of the Cramer structural class data and using those as arguments to compute the CDFs with a sample size of 1,000.

The assumptions of normality were further tested using the Shapiro-Wilk test and by plotting a quantile-quantile plot (qqplot). The goodness of fit from the qqplot provided another means to visually inspect whether the Cramer structural class data for each class were normally distributed.

Inspection of the ECDFs for the 3 Cramer structural classes allowed the level of separation between classes to be assessed visually. A Kolmogorov-Smirnov (*K-S*) test (Conover, 1999) (https://en.wikipedia.org/wiki/Kolmogorov%E2%80%93Smirnov_test) was also performed to verify the difference between distributions.

5th percentiles were derived from the ECDFs and used to calculate the associated TTC values in both mg/m^3^ and ppm units based on the methods described in Escher et al. (2010).


$$\begin{array}{l} Threshold\;\left(TTC\right)=\frac{5thpercentileNOEC\times dexp}{10\times 2.5} \end{array}$$

where dexp is a daily exposure of $\frac{6}{24h}\times \;\frac{5}{7d}$

To convert NOEC/L values in mg/m^3^ to body doses:


$$\begin{array}{l} NOEL\;\left(ug/kg/d\right)=5thpercentileNOEC\frac{mg}{m^{3}}\times dexp\\ \;\times \frac{Vresp\frac{m^{3}}{d}}{bwkg}\times 1000 \end{array}$$

where Vresp, the human respiratory volume is 20 m^3^ for consumers (European Chemicals Agency ECHA, 2008) and bw human body weight is 60 kg per Munro et al. (1996).

### Development of New TTC Values Based on Other Subcategorisations of the Data

Substances that were assigned to one of the Cramer classes, were also profiled on the basis of their MOA for aquatic toxicity using the Verhaar module (Verhaar et al., 1992) within Toxtree v3.1, as well as the Verhaar profiler and the OASIS Mode of action acute toxicity profiler within the OECD Toolbox v4.3. An overall outcome was taken of the 3 profiling outcomes as follows: if the profiling outcomes all agreed, that formed the final outcome else the majority outcome was taken. If the schemes disagreed with each other, the most conservative outcome was taken.

Empirical cumulative distributions were plotted of the NOAEL values in log10(mg/m^3^) to explore whether the overall MOA was a reasonable discriminator for the substances. A Kolmogorov-Smirnov (*K-S*) test (Conover, 1999) was also performed to verify the difference in distributions.

5th percentile values were then bootstrapped using 10,000 replicates and the median and of the bootstrapped 5th percentile distribution was used to derive new TTC values (see notebook 07).

### Verification of Carthew et al. (2009) and Escher et al. (2010) TTC Values

For each dataset, the 5th percentile and TTC values for each Cramer class were calculated individually for local and systemic effects. The data present in the “Local NOAEC (mg/m^3^)” and “Systemic NOAEL (mg/kg/day)” columns were utilized for the Carthew dataset; whilst, for the Escher dataset, the “Local_NOEC_ppm” and “Systemic_NOEC_ppm” columns were used. Additionally, the “General_NOEC_ppm” column from the Escher dataset was utilized to calculate the general 5th percentile and TTC values. As described above, the general NOEC values in the Escher dataset are the lowest NOEC value for a particular substance and, therefore, provide a NOEC where no local or systemic effect would likely be observed. Comparisons of the structural features of the Carthew and Escher datasets relative to the ToxValDB dataset was performed using the ToxPrint chemical fingerprints (Yang et al., 2015) and projecting them into a 2D scatterplot using a t-distributed stochastic neighbor embedding (TSNE) approach, as implemented in the python package scikit-learn, with a learning rate of 200 (see notebook 08).

### Data Analysis Software and Code

Data processing was conducted using the Anaconda distribution of Python 3.6 (Anaconda.org) and associated libraries—scikit-learn, pandas, numpy, visualization tools matplotlib, and seaborn and the statistical library scipy within the Jupyter lab environment. Python Jupyter Notebooks are available on github (at https://github.com/g-patlewicz/inhalation-ttc) and all the datasets are posted on the EPA FTP website (ftp://newftp.epa.gov/Computational_Toxicology_Data/CCTE_Publication_Data/CCED_Publication_Data/PatlewiczGrace/Frontiers-TTC/).

## Results and Discussion

### Results: Profiling of Substances Through the Kroes et al. (2004) Workflow Within Toxtree: ToxValDB

The 4,703 substances with QSAR Ready SMILES were profiled through the Kroes TTC decision tree within Toxtree v3.1. Of the 4,703 structures, 2 substances (both cyclic siloxanes: DTXSID80871154 and DTXSID10870958) could not be processed by the Kroes module. Table 1 shows the assignments of the substances into their specific TTC assignment after processing through the Kroes decision tree, the Cramer rules and the custom profilers from Nelms et al. (2019) and Patlewicz et al. (2018). Although 22% of substances were assigned as flagging an alert for genotoxicity, 72% of the substances were captured by the Cramer structural classes. Similar to other TTC analyses e.g. Munro et al. (1996), there was a paucity of substances assigned to Cramer class II. The set of substances assigned by Cramer structural classes formed the basis of the remainder of the analysis.

**Table 1:** Numbers of chemicals assigned by TTC category.

**TTC category**	**Number of chemicals**
Not processed	2
High potency carcinogens	18
Organophosphates	70
Carbamates	17
Steroids	0
Substances presenting a genetox structural alert	1,077
Substances otherwise not appropriate for TTC	130
Cramer Structural I	1,498
Cramer Structural II	165
Cramer Structural III	1,726

### Processing ToxValDB to Create Cramer Structural Class Datasets

The starting dataset comprised 675,694 records for 4,703 different substances. Subsetting and filtering ToxValDB to identify study records that met certain criteria in terms of study type, units, and tagged by assignment into one of the 3 Cramer class resulted in 1,525 studies for Cramer I, 77 studies for Cramer II, and 1,186 studies for Cramer III substances.

For each structural class, the minimum representative toxicity value (NOAEL/NOAEC) after removal of extreme outliers was identified for each substance in each of Cramer structural classes. There were 244 substances with values for Class I, 14 for Class II and 261 for Class III. The breakdown of chemicals and studies are reflected in Table 2.

**Table 2:** Breakdown of number of studies/chemicals within ToxValDB across the 3 Cramer classes.

**Cramer class**	**Total number of studies**	**No of chemicals with representative study**
I	1,525	244
II	77	14
III	1,186	261

### Derivation of 5th Percentiles for Cramer Structural Classes

**Figure 2:**
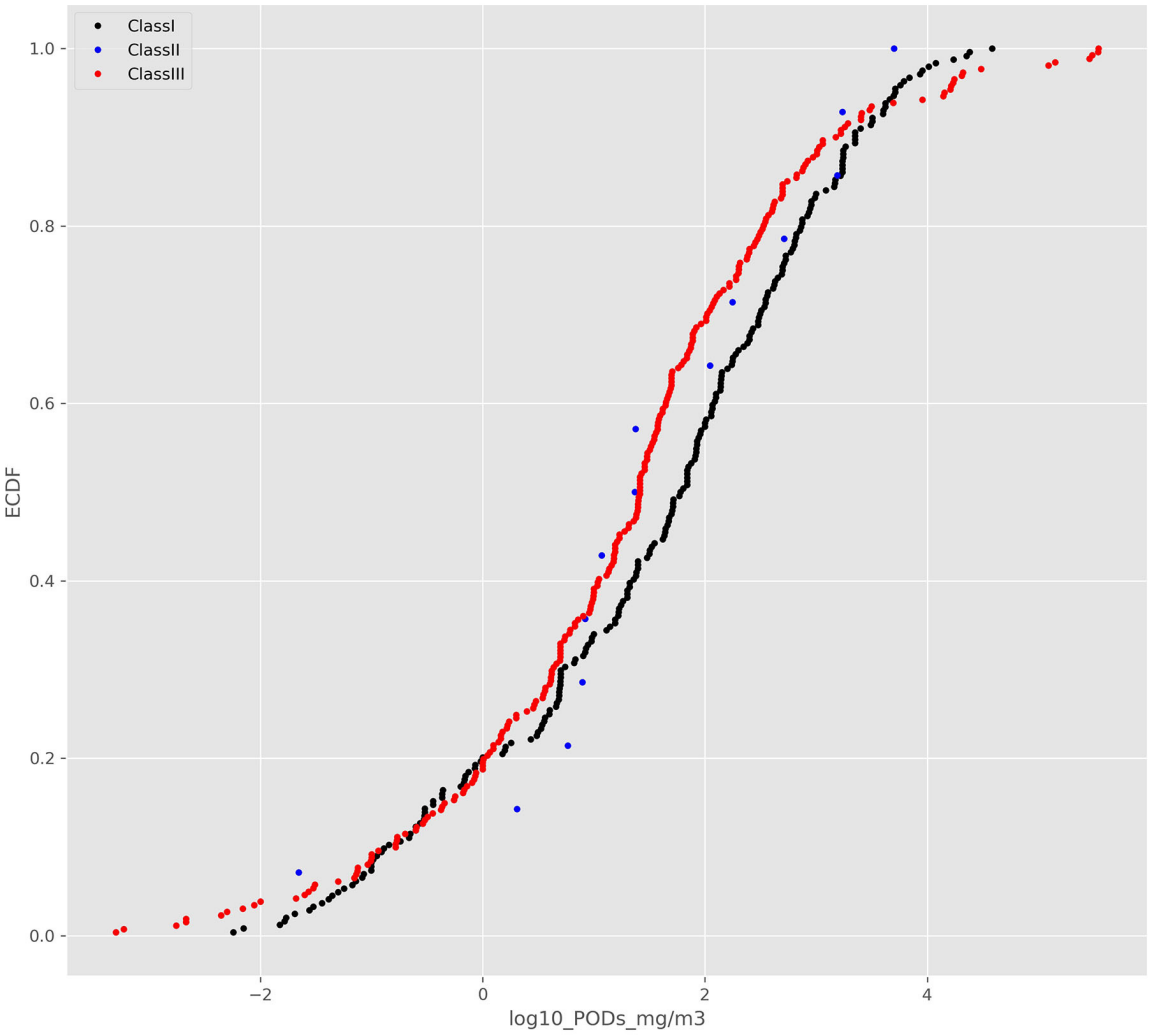
ECDFs of the ToxValDB data as categorized into the 3 Cramer structural classes.

The ECDFs for the 3 structural classes are shown in Figure 2.

Whilst the 2-sample *K-S* test suggested that there was a difference between the Cramer class I and class III distribution (*D* = 0.16, *p* = 0.00254), visual inspection reveals no clear separation between the 3 Cramer classes.

Overlaying CDFs distributions from a normal distribution on the ECDFs suggests that the Cramer structural classes are poorly aligned and not following a normal distribution (Supplementary Figure 1). Further, fitting a normal distribution to the Cramer structural class I or III and plotting qqplots (Supplementary Figure 2, shown for Class I only) as well as computing Wilks-Shapiro tests Shapiro and Wilk (1965) demonstrated that neither Cramer structural class I or III was normally distributed. The Wilks-Shapiro test statistic for Cramer structural class I was 0.971 at a *p* value of 0.00, whereas the test statistic was 0.988 with a *p* value of 0.03 for structural class III. Given the lack of correspondence between the CDFs and ECDFs and the results of the Wilks-Shapiro test, it was concluded that the Cramer structural class distributions were not normally distributed and that computing the 5th percentile from the CDFs was not appropriate.

Rather the 5th percentile from the ECDFs was computed for each of the structural classes using their mg/m^3^ units (Table 3).

**Table 3:** Derived TTC values for the 3 Cramer classes.

**Cramer class**	**Number of chemicals**	**5th percentile (mg/m^**3**^)**	**TTC value (mg/m^**3**^)**	**NOEL (ug/kg/d)**	**TTC (ug/person/d)**
I	244	0.0579	0.000414	3.445	8.271
II	14	0.416	0.002975	24.79	59.507
III	261	0.0299	0.000214	1.786	4.286

The results of Class II contained too few substances to be considered further. The TTC values derived from the ECDFs were compared with the associated values from the Escher et al. (2010) and Carthew et al. (2009) publications.

It is worth noting that the 5th percentiles and their associated TTC values for Cramer I and III are not particularly different, highlighting the lack of separation in the distributions between the 2 classes (see Table 4). In Escher et al. (2010), the general TTC per person and per mg/m^3^ were most comparable to the analysis performed; here, the TTC value for Cramer III was quite comparable to the ToxValDB derived value but there was a much clearer separation between the Cramer structural classes in Escher et al. (2010). For the Carthew et al. (2009) dataset, the systemic TTC values were most meaningful to compare, the published value of 170 ug/person/day and 0.0492 mg/m^3^ were very different to those derived from ToxValDB.

**Table 4:** Comparison of the inhalation results from ToxValDB relative to those reported in Carthew et al. (2009) and Escher et al. (2010).

**Source**	**Number of chemicals**	**Structural class**	**TTC mg/m^**3**^**	**TTC ug/person/d**
ToxValDB	244	Cramer I	4.14E-03	8.27
	14	Cramer II	2.975E-03	59.5
	261	Cramer III	2.14E-04	4.28
Escher et al. (2010)	58	Cramer I	3.6E-03	71
	7	Cramer II	4.8E-04	10
	138	Cramer III	1.8E-04	4
Carthew et al. (2009)	38	Cramer I	0.049	980
	50	Cramer III	8.5E-03	170

**Figure 3:**
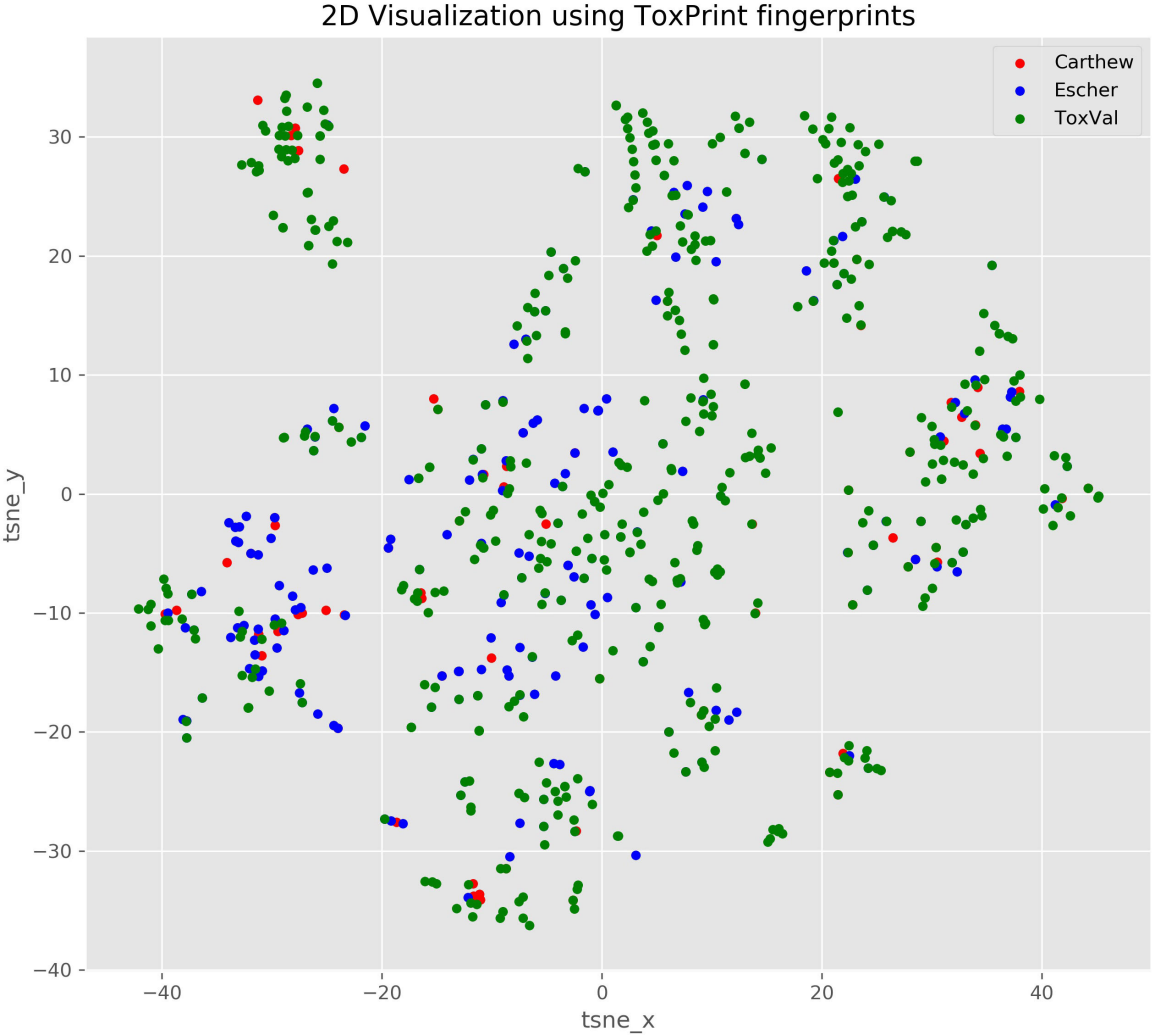
TSNE 2D scatterplot representation of the 3 datasets using ToxPrint chemical fingerprints.

Based on the published figures extracted from the articles, it is evident that the derived TTC values are very different for the Cramer I across the 2 publications and from ToxValDB and that whilst the values are comparable for Cramer Class III between ToxValDB and Escher et al. (2010), they were very different from Carthew et al. (2009). This can be partially explained by the chemical makeup of the respective datasets as evidenced by the 2D structural landscape. The Escher dataset is more broadly represented within the ToxValDB landscape whereas the smaller Carthew dataset is sparsely distributed across the other 2 datasets (see Figure 3).

A comparison of the chemical overlap between the three datasets was performed on the basis of their chemical identifiers using DTXSID. A Venn diagram showing the overlaps is shown in Supplementary Figure 3. A comparison of the overlaps between the 3 datasets were undertaken to compare the actual toxicity values reported to evaluate whether the differences in TTC values were more likely to be as result of differences in the toxicity values.

There were 101 substances in the ToxValDB dataset that overlapped with the Escher dataset but their reported values appeared in many cases quite different. This is reflected by the low pearson correlation coefficient of 0.55. Supplementary Figure 4 shows the scatterplot of the log transformed NOAEC values between the 2 datasets. A boxplot (Supplementary Figure 5) shows the NOAEC distributions of the 2 datasets, a *t*-test performed (*t* = −1.26, *p* = 0.21) reveals that the null hypothesis of their means being the same can not be rejected.

There were 54 substances with data that overlapped the ToxValDB and the Carthew dataset. A boxplot (Supplementary Figure 6) shows the NOAEC distributions of the 2 datasets, a *t*-test performed (*t* = 1.096, *p* = 0.275) reveals that the null hypothesis of their means being the same can not be rejected. However, the pearson correlation coefficient was low at 0.567 (Supplementary Figure 7).

Although chemical landscape between the datasets is one contributing factor to explaining why the TTC values derived could be different, the differences in the toxicity values appears to play a larger role, indeed for substances that were common between the datasets, there was a low agreement between the toxicity values reported.

The lack of separation in the Cramer structural classes and the fact that the data did not fit the expected theoretical distribution prompted further evaluation of the dataset and consideration of another means of subcategorizing the chemicals.

### Reproduction of the TTC Values Reported by Carthew et al. (2009) and Escher et al. (2010)

For the Escher et al. (2010), it was assumed that the percentiles derived from the different datasets were calculated directly from the experimental data rather than from fitting a theoretical distribution. TTC values for the Cramer I and III classes were derived using the General_NOEC_ppm data provided in the Supplementary Information of Escher et al. (2010) (Table 5).

**Table 5:** Reported and rederived TTC values of the Escher et al. (2010) and Carthew et al. (2009) studies.

**Structural category**	**Reported #chemicals**	**Reported TTC mg/m^**3**^**	**Reported TTC ug/person/d**	**Reproduced #chemicals**	**Reproduced TTC mg/m^**3**^**	**Reproduced TTC ug/person/d**
Escher et al. (2010)						
Cramer I	58	3.6E-03	71	58	4.57E-03	91.44
Cramer III	138	1.8E-04	4	137^*^	2.78E-04	5.56
Carthew et al. (2009)						
Cramer I	38	0.049	980	38	0.043	865
Cramer III	50	8.5E-03	170	50	7.2E-03	145

* *One of the NOEC values reported was 0 which was dropped from consideration*.

On the basis of the information presented in the original publication, it was not possible to replicate the General TTC values reported in Table 1 of the Escher et al. (2010) publication.

For the Carthew et al. (2009) dataset, it was assumed that the percentiles were derived from the experimental data rather than a fitted theoretical distribution. Systemic NOAEL values in mg/kg/day were first converted into mg/m^3^. Therein, TTC values in mg/m^3^ and ug/person/d were reproduced and compared with reported values (Table 5).

TTC values as reported in the original articles could not be exactly reproduced based on the information provided for the systemic endpoints.

### Proposing New TTC Values Using Acute Aquatic Mode of Action Profilers to Categorize to the ToxValDB

Plots of the ECDF for OASIS MOA and Verhaar scheme as computed by the OECD Toolbox and the Verhaar scheme from Toxtree were plotted to explore the separation in the distributions (Supplementary Figures 8A,B). There appeared to be a good separation between baseline and reactive MOAs. Other ECDFs were less pronounced owing the sparsity of study outcomes for esters, narcotic amine and the other MOAs which comprised phenols and anilines, aldehydes and alpha, beta-unsaturated alcohols. Good separation was also noted in the distributions when profiled by the Toxtree Verhaar scheme (Supplementary Figure 9). The number of chemicals in each category are provided in Supplementary Table 1 for all the profilers. In each case, there was good separation in the distributions of baseline assigned substances from the other MOA outcomes, notably those substances assigned as reactive. A “consensus” outcome of the MOA assignments were derived by combining the Toolbox and Toxtree outcomes for subsequent TTC derivation.

Since the Verhaar scheme was computed by both tools, a comparison of their concordance was first performed. Supplementary Figure 10 shows the confusion matrix comparing the Toxtree and Toolbox Verhaar designations.

There were a number of mis-classifications between the 2 Verhaar schemes—though the most consistent assignments were for baseline narcotics. The precision and recall for the Toolbox Verhaar assigning baseline narcotics as such by the Toxtree Verhaar was 90 and 76%, respectively. The precision for Toolbox Verhaar assigning substances as Toxtree Verhaar reactives was 31%, whereas the recall was 71%. An overall outcome was created to combine the 3 schemes into one “consensus” MOA designation that reflected the most conservative assignment.

**Figure 4:**
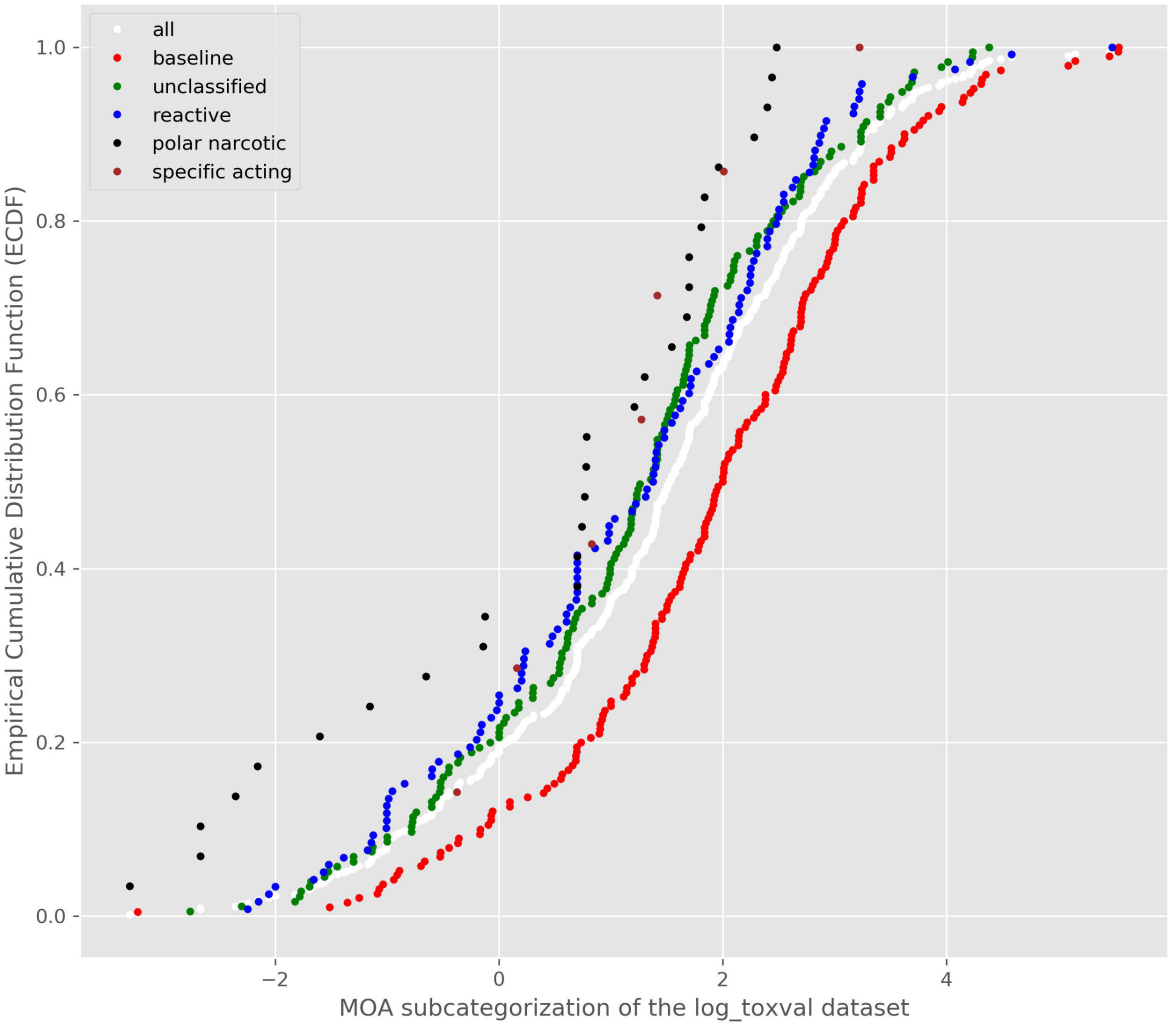
ECDF of the consensus MOA assigned to each substance.

The combined MOA assignment from the 3 schemes and plotting their ECDFs is shown in Figure 4.

There appeared to be good separation of the baseline narcotics from the other MOAs and the reactive toxicity distribution appeared to follow the unclassfied distribution. A *K-S* test was conducted and confirmed the differences between the reactive and baseline narcotics distributions (*D* = 0.22, *p* = 0.0013). Accordingly, bootstrapped replicates were created to derive a bootstrapped 5th percentile of the baseline and reactive toxicity distributions. The median of all the 5th percentile distributions was carried forward into the TTC derivations. Table 6 summarizes the TTC values derived for the baseline and reactive toxicity categories.

**Table 6:** TTC values derived from the baseline narcotic and reactive bootstrapped 5th percentiles.

**Classification**	**#Chemicals**	**5th percentile median bootstrapped**	**TTC mg/m^**3**^**	**TTC ug/person/d**
Baseline	190	0.1567	1.11E-03	22.39
Reactive	118	0.0299	2.14E-04	4.286

## Conclusions

TTCs are well-established for the oral route of entry but less attention has been spent investigating the feasibility of deriving TTC values for the inhalation route of exposure. Here, an effort was made to derive new TTC thresholds using data collected within the ToxValDB. The chemicals in the dataset were profiled in the Kroes et al. (2004) workflow as adapted in Patlewicz et al. (2018) and Nelms et al. (2019). TTC values were derived using the 5th percentiles for the empirical cumulative distributions as the distributions did not fit a normal distribution on the basis of their toxicity values [transformed to log10(mg/m^3^)]. The values were compared to those previously published by Carthew et al. (2009) and Escher et al. (2010). TTC values were similar for the Cramer Class III with the Escher et al. (2010) published value, but there was little correspondence between Escher et al. (2010) and Carthew et al. (2009) and those values derived from ToxValDB. There was no good separation between the Cramer classes within the ToxValDB and the TTC values derived were only considered relevant from a comparison basis. A combination of the structural differences between the datasets as well as differences in the toxicity values reported for overlapping chemicals is thought to explain the lack of separation by using the Cramer structural classes and in the TTC values themselves. Further evaluation of the studies reported in ToxValDB is merited and will be the subject of ongoing research.

The Carthew and Escher datasets were then evaluated to determine whether the published values could be reproduced based on the information reported. Although the values derived were similar, the 5th percentiles and TTC values were not the same as what was reported. This is thought to be in part due to rounding errors, an inability to identify all chemicals in the respective datasets and the lack of specific details provided in the articles of the methods undertaken.

In light of the Cramer classes not forming a good means of differentiating categories of chemicals for the ToxValDB dataset, a MOA profiling scheme derived for aquatic toxicity was used to profile the chemicals into classes. This was chosen based on work by Veith et al. (2009) who determined that there was a correlation between acute inhalation and acute aquatic toxicity modes of action. Veith et al. (2006) had also established that substances that were reactive correlated well with acute inhalation toxicity. Accordingly, aquatic fish toxicity mode of action structural schemes were used to profile the dataset and ECDFs were drawn to explore the separation of toxicity across the different classes. Since more than one approach is available to profile for aquatic toxicity a consensus outcome was used to categorize substances. There was a clear separation between the reactive and baseline distributions as verified from the ECDFs and on the basis of the *K-S* test. Since the data was not normally distributed, bootstrapping was used to compute a distribution of 5th percentile values using 10,000 bootstrapped replicates. The median of this bootstrapped replicate distribution was used to compute the associated TTC values. The reactive TTC value was comparable to that of the Cramer structural classes, a more conservative TTC value was determined for the baseline category.

These new inhalation TTC thresholds (22.4 ug/person/day for baseline narcotics and 4.3 ug/person/day for reactive toxicants) could complement established oral TTC thresholds for the purposes of facilitating the risk-based prioritizations of thousands of substances lacking empirical data.

## Data Availability Statement

All datasets are available at ftp://newftp.epa.gov/Computational_Toxicology_Data/CCTE_Publication_Data/CCED_Publication_Data/PatlewiczGrace/Frontiers-TTC/. Code is available at https://github.com/g-patlewicz/inhalation-ttc.

## Author Contributions

MN conducted initial analysis of structuring datasets, performing preliminary analysis, and drafting part of Methods section. GP conducted remaining analysis and drafting of manuscript.

## Conflict of Interest

The authors declare that the research was conducted in the absence of any commercial or financial relationships that could be construed as a potential conflict of interest.

## Supplementary Material

Supplementary Figure 1 CDFs and ECDFs for the Cramer structural classes.

Supplementary Figure 2 qqplots for Cramer Class I.

Supplementary Figure 3 Venn diagram showing overlap of chemicals between the ToxValDB Cramer dataset and the Escher and Carthew datasets.

Supplementary Figure 4 Scatterplot of ToxValDB and Escher toxicity values.

Supplementary Figure 5 Boxplot of ToxValDB and Escher toxicity values.

Supplementary Figure 6 Boxplot of ToxValDB and Carthew toxicity values.

Supplementary Figure 7 Scatterplot of ToxValDB and Carthew toxicity values.

Supplementary Figure 8 **(A)** ECDFs for substances profiled through the OASIS MOA acute toxicity profiler. **(B)** ECDFs for substances profiled through the Toolbox Verhaar MOA profiler.

Supplementary Figure 9 ECDF of substances profiled by the Toxtree Verhaar scheme.

Supplementary Figure 10 Confusion matrix for the Verhaar assignments from the Toolbox and Toxtree.

Supplementary Table 1 Chemical counts per MOA assignment per profiler.

Supplementary Data File 1 Processed Escher et al. (2010) dataset.

Supplementary Data File 2 Processed Carthew et al. (2009) dataset.
