# Supplementary figures and images for: Derivation of New Threshold of Toxicological Concern Values for Exposure via Inhalation for Environmentally-Relevant Chemicals
Source: Front Toxicol. 2020 Oct 16;2:580347. doi: 10.3389/ftox.2020.580347 (PMC8915872; doi:10.3389/ftox.2020.580347)

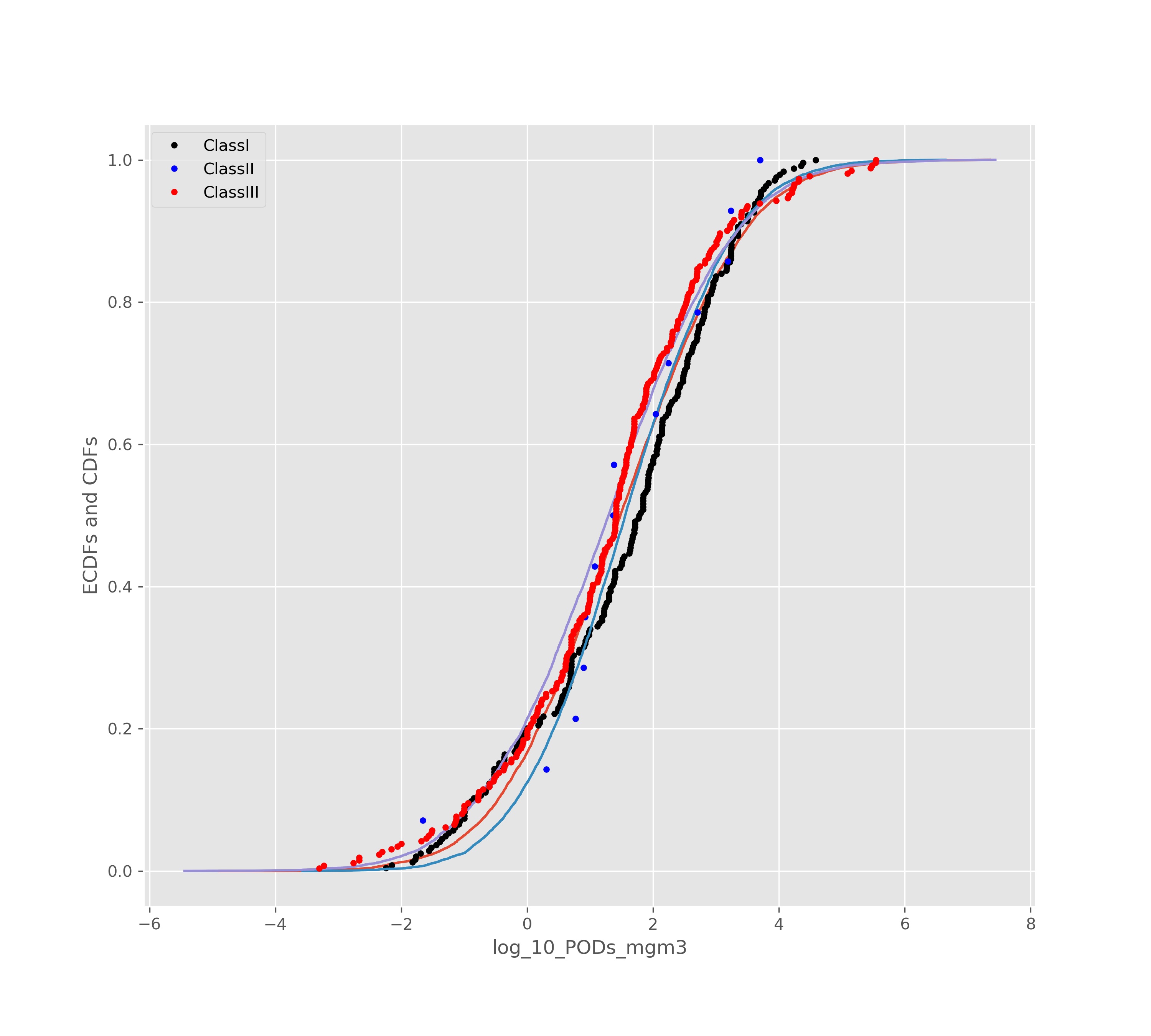

Supplement: Supplementary Figure 1 — CDFs and ECDFs for the Cramer structural classes. [file Image_1.jpg]

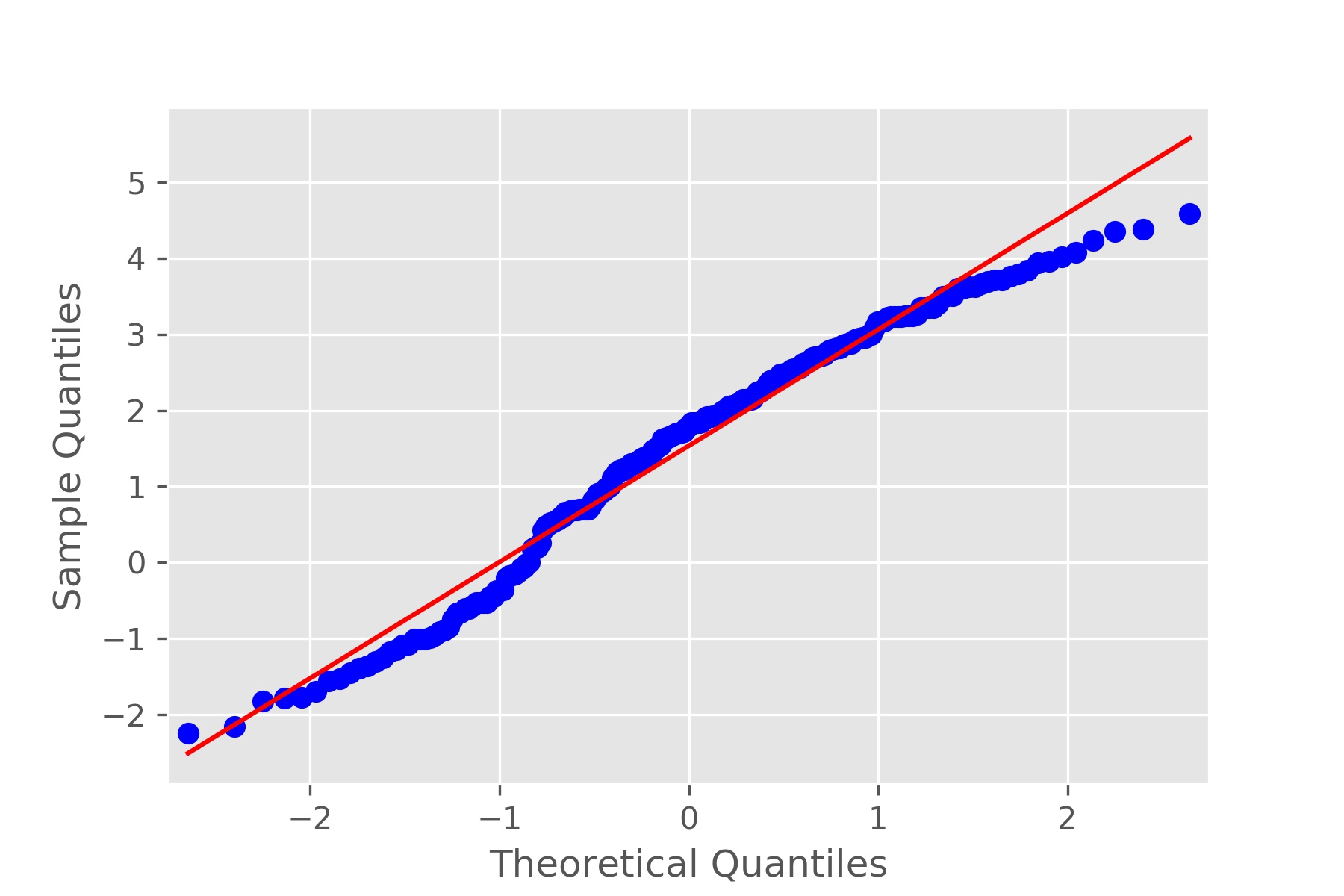

Supplement: Supplementary Figure 2 — qqplots for Cramer Class I. [file Image_2.jpg]

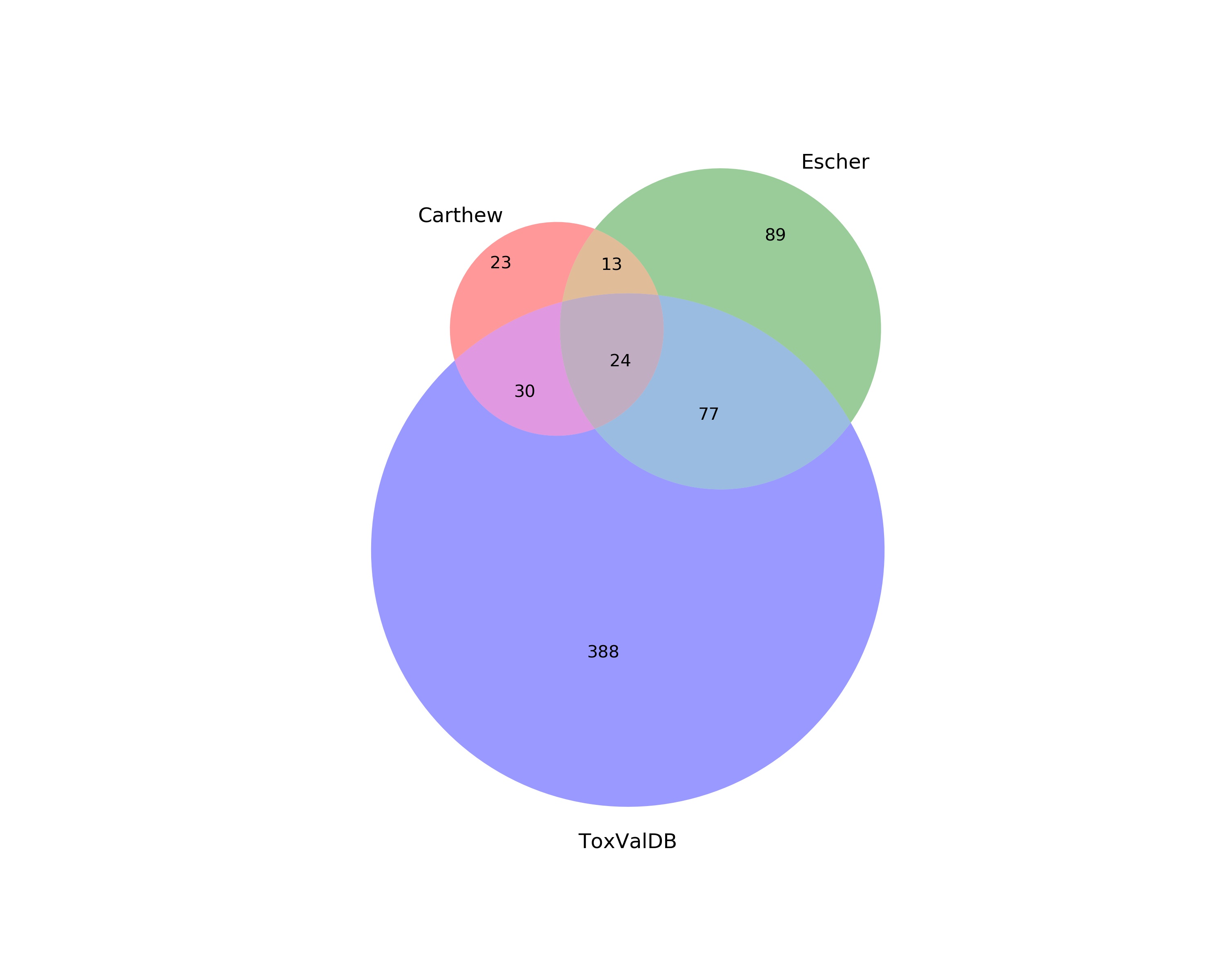

Supplement: Supplementary Figure 3 — Venn diagram showing overlap of chemicals between the ToxValDB Cramer dataset and the Escher and Carthew datasets. [file Image_3.jpg]

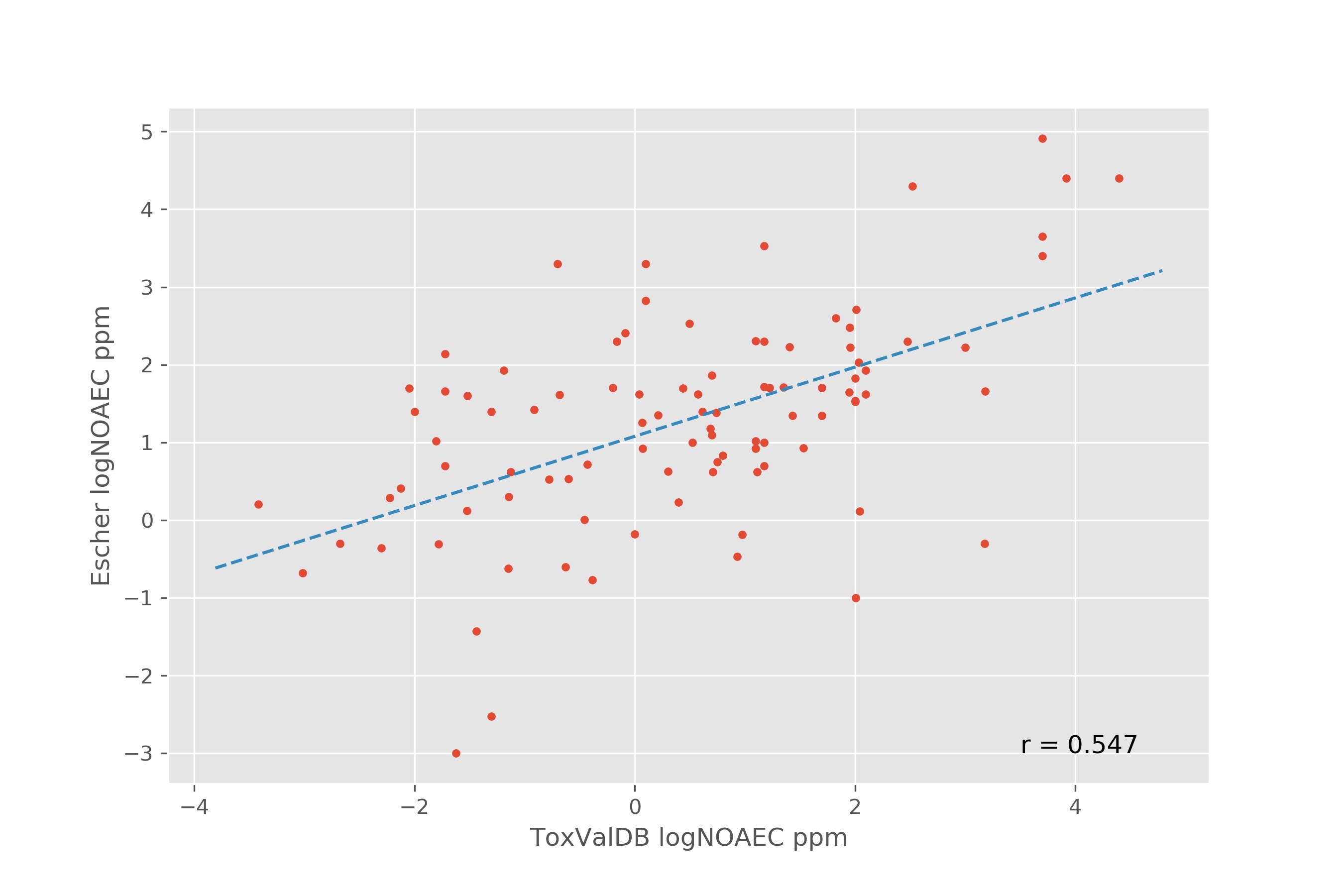

Supplement: Supplementary Figure 4 — Scatterplot of ToxValDB and Escher toxicity values. [file Image_4.jpg]

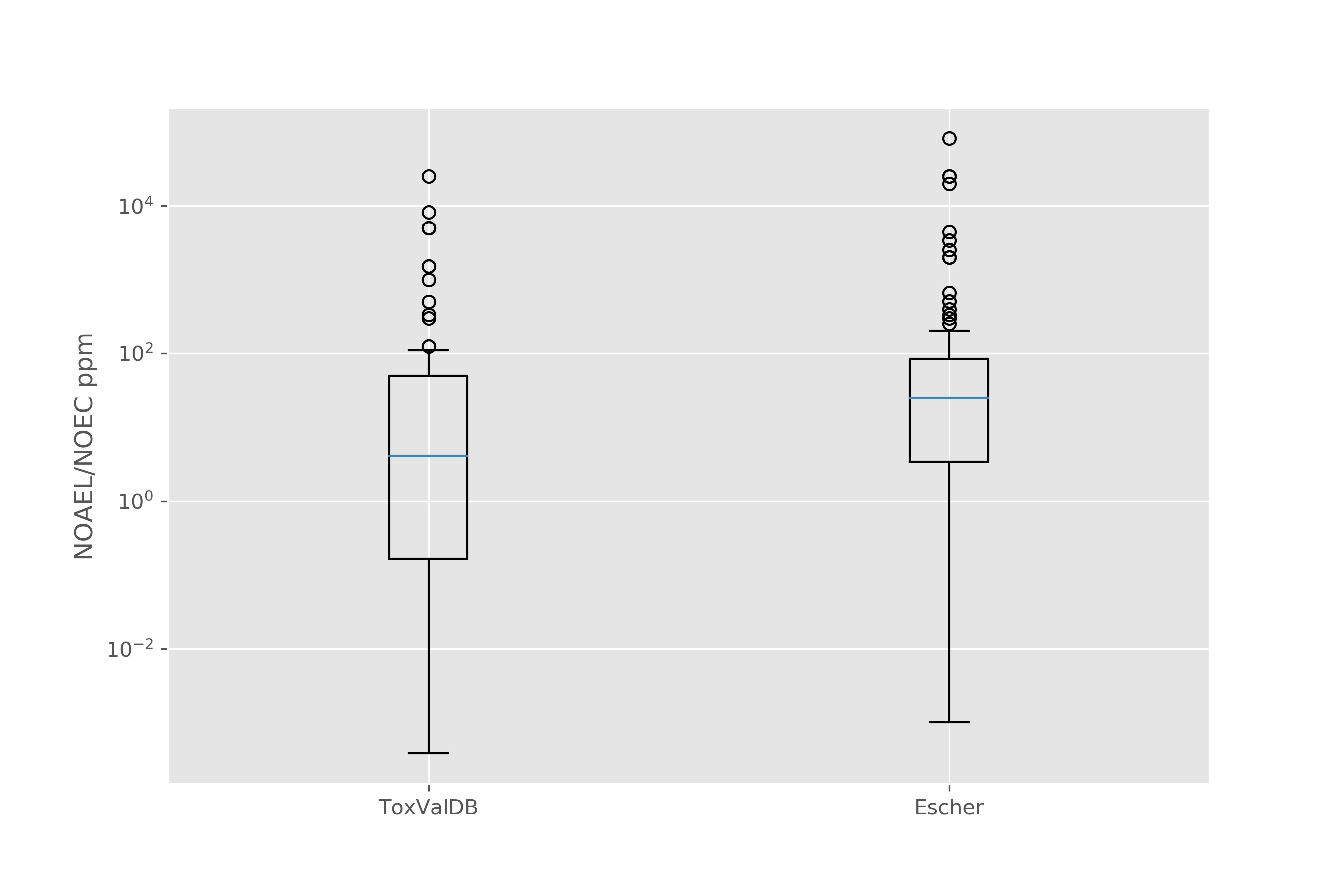

Supplement: Supplementary Figure 5 — Boxplot of ToxValDB and Escher toxicity values. [file Image_5.jpg]

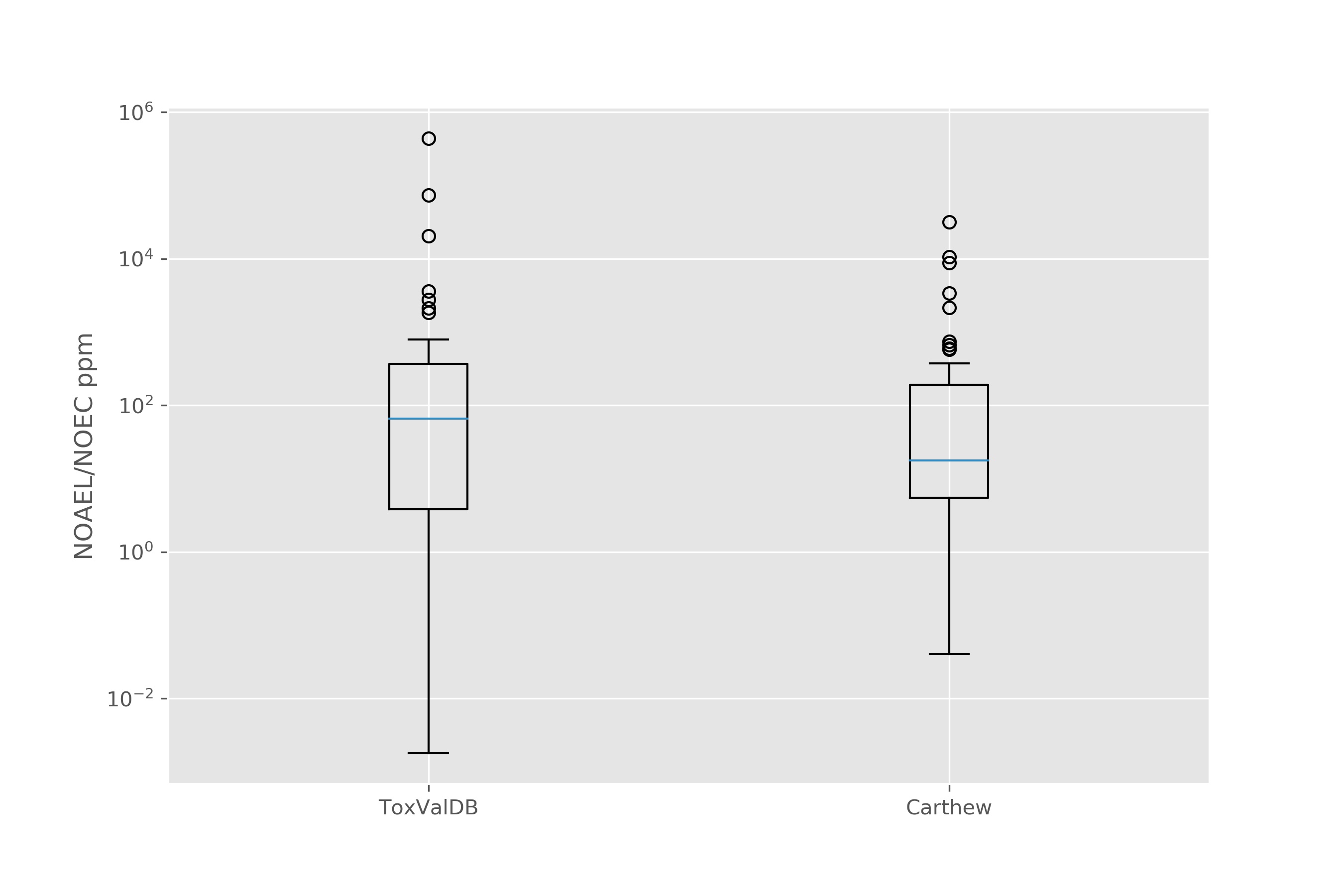

Supplement: Supplementary Figure 6 — Boxplot of ToxValDB and Carthew toxicity values. [file Image_6.jpg]

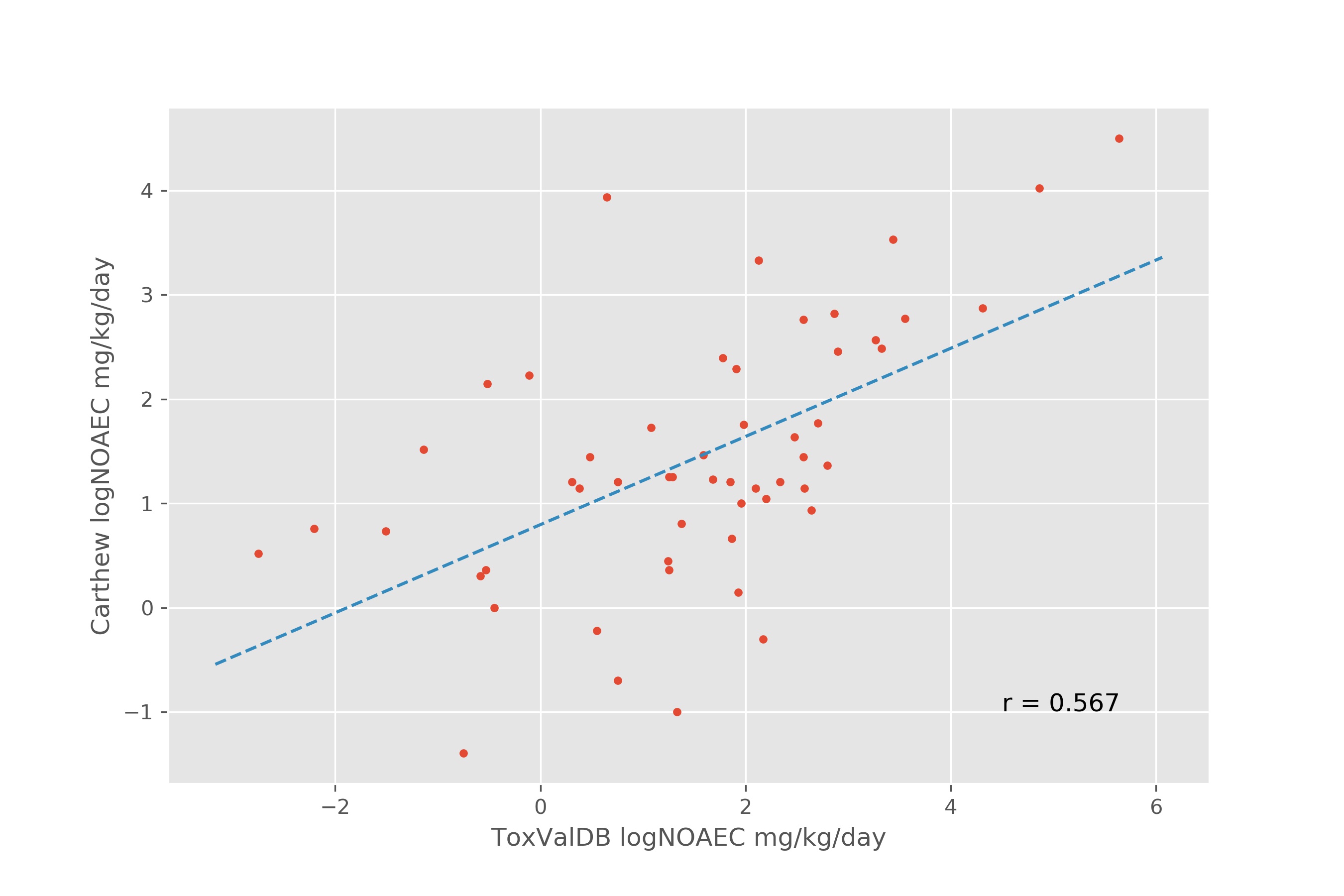

Supplement: Supplementary Figure 7 — Scatterplot of ToxValDB and Carthew toxicity values. [file Image_7.jpg]

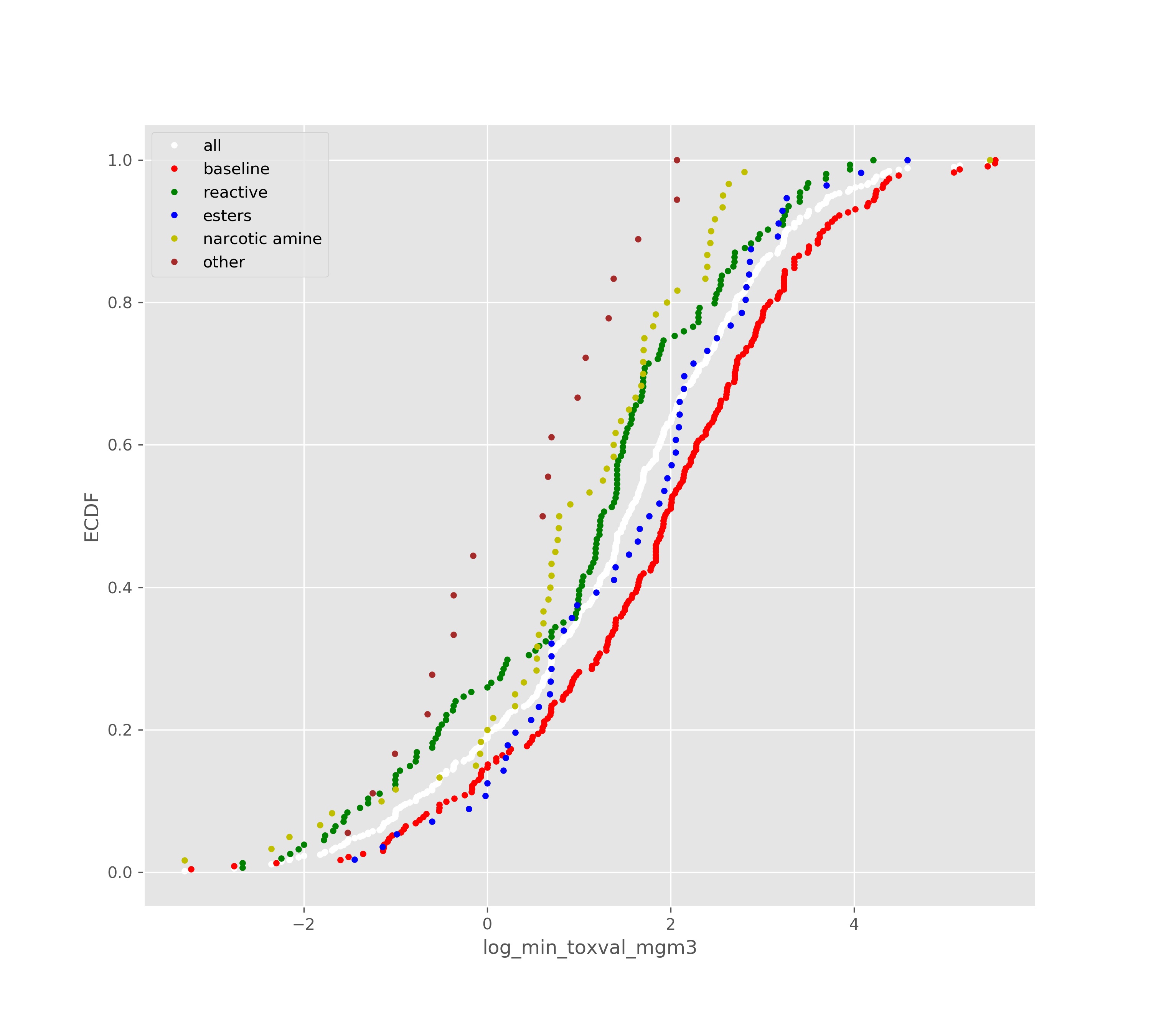

Supplement: Supplementary Figure 8 — (A) ECDFs for substances profiled through the OASIS MOA acute toxicity profiler. (B) ECDFs for substances profiled through the Toolbox Verhaar MOA profiler. [file Image_8.jpg]

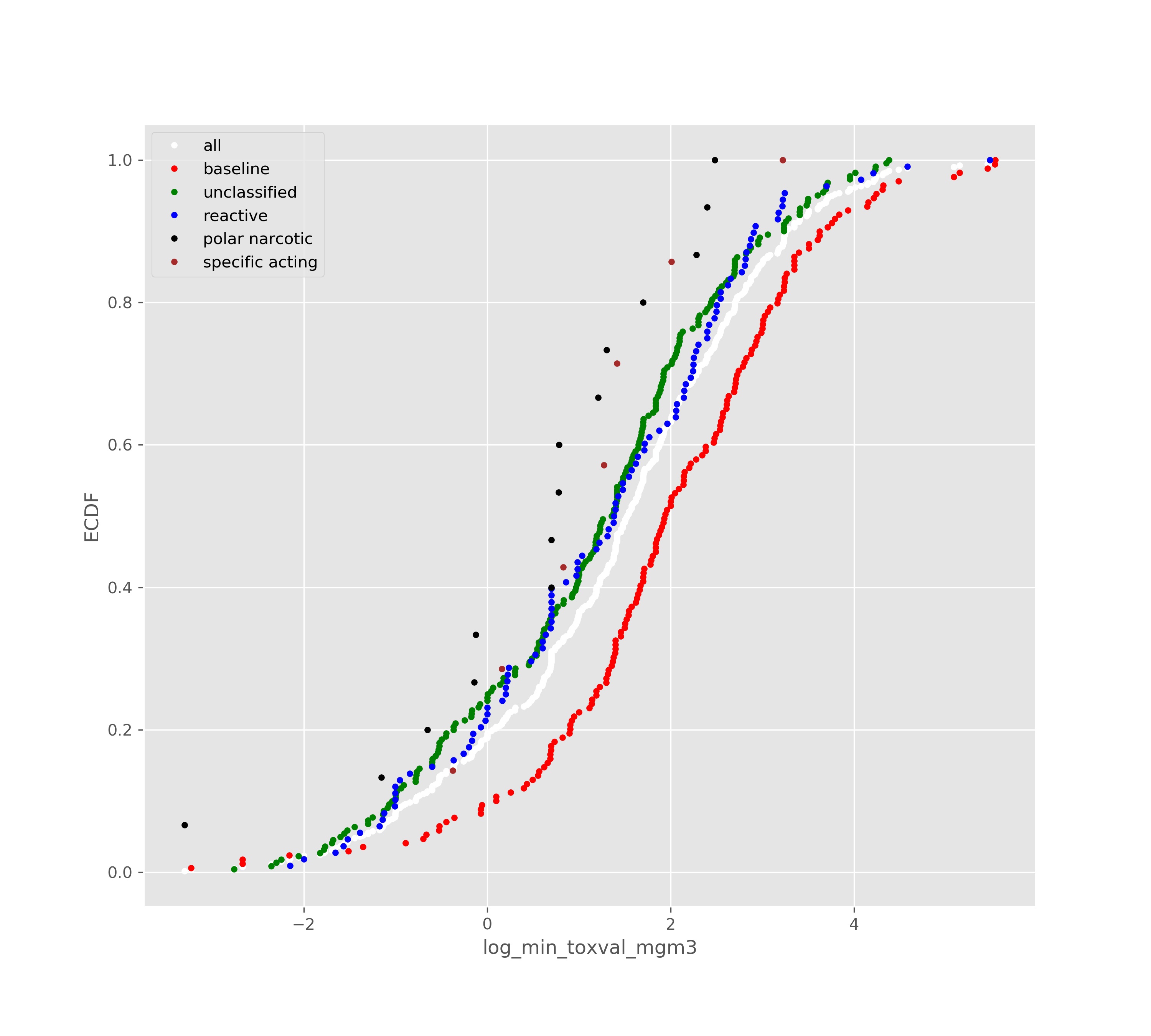

Supplement: Supplementary Figure 9 — ECDF of substances profiled by the Toxtree Verhaar scheme. [file Image_9.jpg]

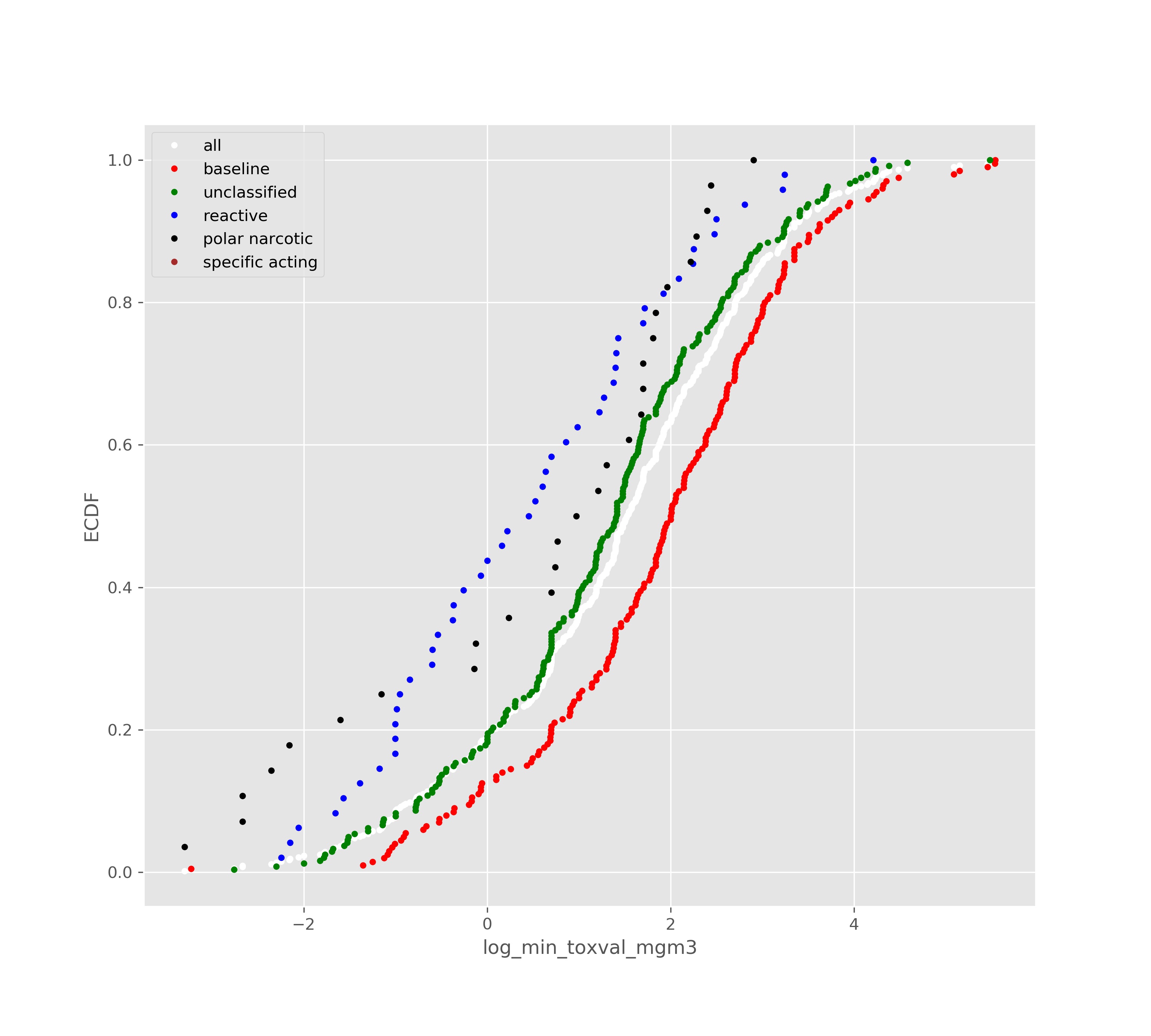

Supplement: Supplementary Figure 10 — Confusion matrix for the Verhaar assignments from the Toolbox and Toxtree. [file Image_10.jpg]

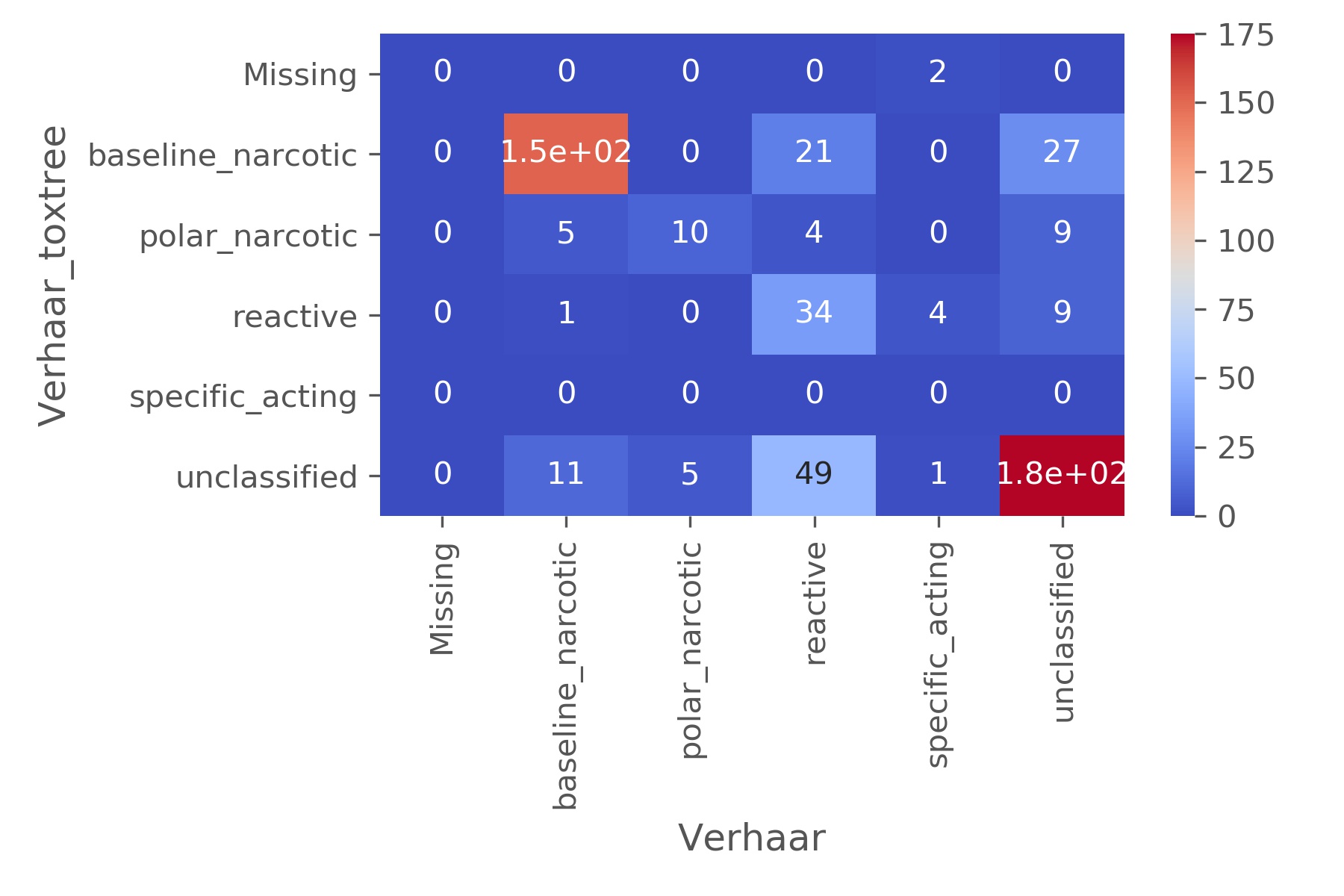

Supplement: Supplementary file 20 [file Image_11.jpg]
