# Supplementary material for: Derivation of New Threshold of Toxicological Concern Values for Exposure via Inhalation for Environmentally-Relevant Chemicals
Source: Front Toxicol. 2020 Oct 16;2:580347. doi: 10.3389/ftox.2020.580347 (PMC8915872; doi:10.3389/ftox.2020.580347)
Supplement: Supplementary Table 1 — Chemical counts per MOA assignment per profiler. [file Table_1.docx]

Table 1 Chemical counts per MOA assignment per profiler

| **MOA Profiler** | **#Chemicals** | **MOA Tool** |
| --- | --- | --- |
| baseline_narcotic | 231 | OASIS MOA |
|  | 169 | Verhaar (Toolbox) |
|  | 200 | Verhaar (Toxtree) |
| reactive | 154 | OASIS MOA |
|  | 108 | Verhaar (Toolbox) |
|  | 48 | Verhaar (Toxtree) |
| narcotic_amine | 60 | OASIS MOA |
| esters | 56 | OASIS MOA |
| polar_narcotic | 15 | Verhaar (Toolbox) |
|  | 28 | Verhaar (Toxtree) |
| specific_acting | 7 | Verhaar (Toolbox) |
| other | 18 | OASIS MOA |
| unclassified | 220 | Verhaar (Toolbox) |
|  | 241 | Verhaar (Toxtree) |
